# Poynting vector analysis for wireless power transfer between magnetically coupled coils with different loads

**DOI:** 10.1038/s41598-017-00846-w

**Published:** 2017-04-07

**Authors:** Yunsheng Guo, Jiansheng Li, Xiaojuan Hou, Xiaolong Lv, Hao Liang, Ji Zhou, Hongya Wu

**Affiliations:** 1 Department of Applied Physics, Inner Mongolia University of Science and Technology, Baotou, 014010 China; 2 School of Information Engineering, Inner Mongolia University of Science and Technology, Baotou, 014010 China; 3 State Key Laboratory of New Ceramics and Fine Processing, School of Materials Science and Engineering, Tsinghua University, Beijing, 100084 China; 4 School of Materials Science and Engineering, Shijiazhuang Tiedao University, Shijiazhuang, 050043 China

## Abstract

Wireless power transfer is a nonradiative type of transmission that is performed in the near-field region. In this region, the electromagnetic fields that are produced by both the transmitting and receiving coils are evanescent fields, which should not transmit energy. This then raises the question of how the energy can be transferred. Here we describe a theoretical study of the two evanescent field distributions at different terminal loads. It is shown that the essential principle of wireless energy transfer is the superposition of the two evanescent fields, and the resulting superimposed field is mediated through the terminal load. If the terminal load is either capacitive or inductive, then the superimposed field cannot transfer the energy because its Poynting vector is zero; in contrast, if the load is resistive, energy can then be conveyed from the transmitting coil to the receiving coil. The simulation results for the magnetic field distributions and the time-domain current waveforms agree very well with the results of the theoretical analysis. This work thus provides a comprehensive understanding of the energy transfer mechanism involved in the magnetic resonant coupling system.

## Introduction

The feasibility of efficient wireless power transfer (WPT) via coupled magnetic resonances has previously been demonstrated both experimentally^1–3^ and theoretically^3–5^. The method used in these studies is based on the principle of resonant evanescent field (either near-field or nonradiative field) coupling, which allows the energy to be coupled efficiently between two resonant objects, i.e., between the transmitting and receiving coils^6^ of the WPT system. Additionally, the coupling mechanism is mediated through overlapping of the evanescent fields^4^. However, in the evanescent electromagnetic field that is generated by a current-carrying coil, the phase difference between the magnetic field ***H*** and the electric field ***E*** is 90°; therefore, the average value of the energy flow density in a single cycle is zero, because the energy is exchanged back and forth in the near field. This means that there is no net energy flow from the transmitting coil to the receiving coil. If this is correct, then no energy transmission should be observed. It is therefore necessary to clarify how the energy is transferred and how the superposition of the evanescent fields mediates the coupling mechanism. While the WPT mechanism has been studied extensively in previous works based on coupled-mode theory^3, 4, 7–9^ and equivalent circuit theory^6, 10–12^, little attention has been paid to the issue of superposition of the evanescent fields, which is related to the essence of the energy transmission process.

In this work, we first establish a simple model of the magnetic resonance-coupled WPT system. Then, the distributions of the incident and reflected fields that are produced by the transmitting and receiving coils, respectively, are depicted under the approximate working conditions, and the effects of the nature (resistive, capacitive or inductive) of the terminal load on the two evanescent electromagnetic fields and the superposition of these fields are discussed. Finally, through analysis of the Poynting vector of the superimposed field at the different types of terminal load, the energy transmission mechanism is clarified.

## Results

### Theoretical model and analysis

**Figure 1:**
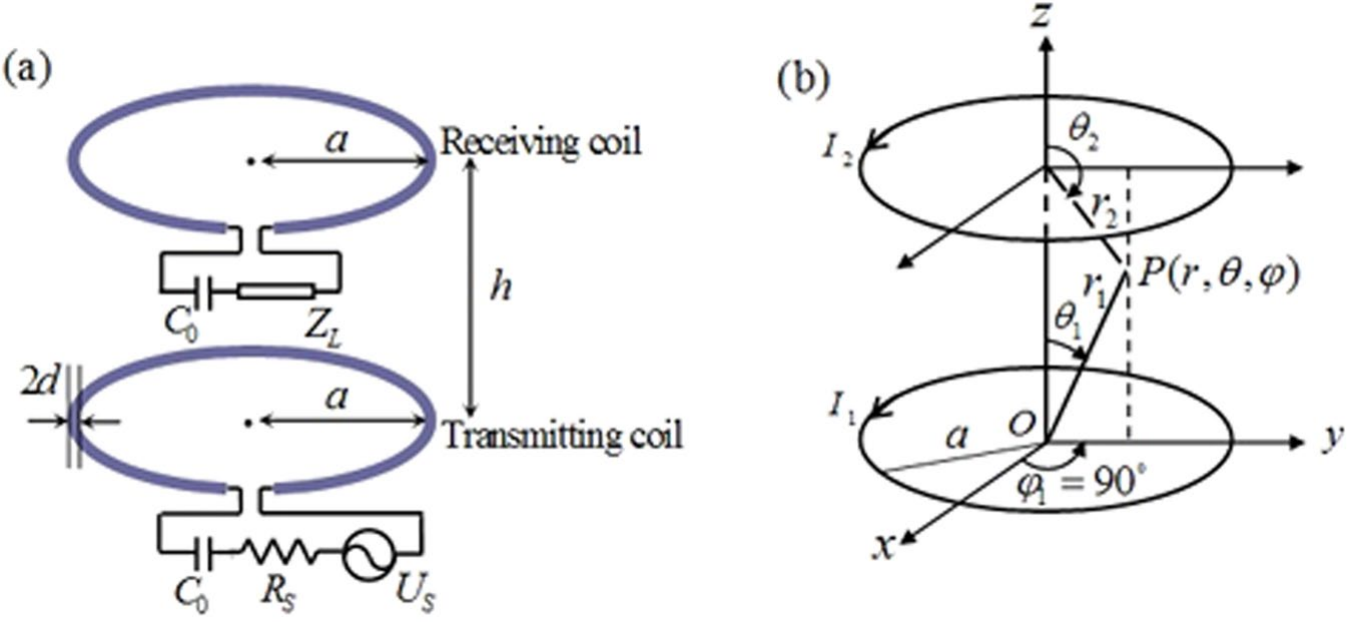
Theoretical model of WPT system. (**a**) Equivalent circuit model. (**b**) Equivalent magnetic dipole model.

The circuit model used for the WPT system^13^ is shown in Fig. 1(a). The transmitting and receiving coils are identical and are aligned coaxially. Each coil is made from a wire with cross-sectional radius *d* that is wound into one loop of radius *a*. The wire is assumed to be a perfect electric conductor (PEC), and thus ohmic loss can be disregarded. A capacitor (*C*_0_) is connected to the coil to achieve resonance. A power source with an electromotive force *U*_*S*_ and an internal resistance *R*_*S*_ is connected to the transmitting coil. A terminal load, for which the impedance *Z*_*L*_ may be inductive, capacitive or resistive, is connected to the receiving coil. The distance between two coils is designated *h*, and represents the transmission distance.

According to Faraday’s law of induction, the time-varying current *I*_1_ that is excited in the transmitting coil by the power source *U*_*S*_ with time-harmonic excitations will induce a time-varying current *I*_2_ in the receiving coil. The two resonant coils are approximated as two current loops and are thus theoretically equivalent to two magnetic dipoles, and this allows the field distributions between them to be calculated. In reality, the effects of the electric dipole that arises from the accumulation of macroscopic charges in the field distributions (mainly in the electric field distribution) should be considered. However, these effects can be negligible because almost the entire electric field is inside the capacitor *C*_0_. In the case where the magnetic coupling only is considered, the near magnetic and electric fields that are generated by either magnetic dipole, using the spherical coordinates shown in Fig. 1(b), are ref. 14

1 $${{\boldsymbol{H}}}_{m}=\frac{{I}_{m}A}{4{\rm{\pi }}}[{{\boldsymbol{r}}}_{0}\frac{2\,\cos \,{\theta }_{m}}{{r}_{m}^{3}}+{{\boldsymbol{\theta }}}_{0}\frac{\sin \,{\theta }_{m}}{{r}_{m}^{3}}],$$

2 $${{\boldsymbol{E}}}_{m}=-{\rm{j}}{{\boldsymbol{\phi }}}_{0}\eta \frac{{I}_{m}Ak}{4{\rm{\pi }}{r}_{m}^{2}}\,\sin \,{\theta }_{m},$$

where *A* (*A* = π*a* ^2^) is the coil area, *k* = 2π*λ* (where *λ* is the wavelength at the operating frequency in free space) is the wave number, $\eta =\sqrt{{\mu }_{0}/{\varepsilon }_{0}}$ is the wave impedance in free space, ${\rm{j}}=\sqrt{-1}$ is the imaginary unit, and the indices *m* = 1 and *m* = 2 denote the incident and reflected fields, respectively. Note that the magnetic and electric fields that are expressed in equations (1) and (2) do not strictly represent the evanescent fields because they do not decrease exponentially with increasing distance. They are actually localized slowly-evanescent fields^4^ and we refer to them as evanescent fields for simplicity. In equations (1) and (2), the phase difference between the magnetic field ***H***_m_ and the electric field ***E***_m_ is 90°, and thus the time-averaged Poynting vector is ref. 14

3 $${{\boldsymbol{S}}}_{m}={\rm{Re}}({{\boldsymbol{E}}}_{m}\times {{\boldsymbol{H}}}_{m}^{\ast })/2=0,$$

where Re indicates the “real part” and the asterisk represents the “complex conjugate.” Equation (3) shows that the energy flow density is zero for the incident and reflected fields, therefore indicating that these evanescent fields cannot transfer energy.

The characteristics of the incident and reflected fields were analysed above; however, the relationship between these fields was not considered in the analysis. In fact, the two fields have a definite relationship that is determined by the electrical properties of the terminal load. In Fig. 1(a), the properties of the terminal load not only affect current *I*_2_ but also affect current *I*_1_. If the current in the transmitting coil is always expressed as *I*_1_ at different terminal loads, then the electric field ***E***_1_ excited by this current can be calculated using equation (4), and the electromotive force induced across the receiving coil is then written as

4 $$V={\int }_{l}{{\boldsymbol{E}}}_{1}\cdot {\rm{d}}{\boldsymbol{l}}=-{\rm{j}}\frac{{I}_{1}\eta {\rm{\pi }}k{a}^{4}}{2{h}^{3}}=-{\rm{j}}F{I}_{1},$$

where d***l*** is the differential length of the receiving coil, and *F* is a constant composed of *η*π*ka* ^4^/(2*h* ^3^). Note that equation (4) is obtained using the condition that *a* ≪ *h*, which is entirely in line with the requirements of the point magnetic dipole model shown in Fig. 1(b). However, this does not mean that the two coils can only be applied within the range where *a* ≪ *h* to transfer energy. Conversely, they can be applied in any near field range. The point magnetic dipole model is introduced here to simplify the calculation of the near magnetic and electric fields.

When the operating frequency (*f*_0_) is equal to the circuit resonant frequency, the impedances of the coil inductance and the capacitance *C*_0_ are offset. Therefore, the current *I*_2_ in the receiving coil is entirely determined by the terminal load, and is given by

5 $${I}_{2}=-{\rm{j}}\frac{F}{{Z}_{L}}{I}_{1},$$

where *Z*_*L*_ = j2π*f*_0_ *L*, *Z*_*L*_ = 1/(j2π*f*_0_ *C*) or *Z*_*L*_ = *R* when the terminal load is an inductor (*L*), capacitor (*C*) or resistor (*R*), respectively. In equation (5), if the terminal load is an inductor, then *I*_2_ and *I*_1_ are out of phase; if the terminal load is a capacitor, then *I*_2_ and *I*_1_ are in phase; and if the terminal load is a resistor, the phase of *I*_2_ lags behind that of *I*_1_ by 90°. Because the field distributions are determined by these currents, the reflected and incident fields have a similar relationship to that of the currents *I*_2_ and *I*_1_. To analyse the Poynting vector of the superimposed fields, an arbitrary point *p* in the *yoz* plane (where *φ*_1_ = *φ*_*2*_ = 90°) between the two magnetic dipoles shown in Fig. 1(b) was selected. Each parameter value of the point *p* in the two spherical coordinates has been marked. Based on equations (1) and (2), the magnetic and electric components of the incident and reflected fields can then be written as

6 $${{\boldsymbol{H}}}_{m}=\frac{{I}_{m}A}{4{\rm{\pi }}}[(\sin \,{\theta }_{m}{{\boldsymbol{y}}}_{0}+\,\cos \,{\theta }_{m}{{\boldsymbol{z}}}_{0})\frac{2\,\cos \,{\theta }_{m}}{{r}_{m}^{3}}+(\cos \,{\theta }_{m}{{\boldsymbol{y}}}_{0}-\,\sin \,{\theta }_{m}{{\boldsymbol{z}}}_{0})\frac{\sin \,{\theta }_{m}}{{r}_{m}^{3}}],$$

7 $${{\boldsymbol{E}}}_{m}={\rm{j}}{x}_{0}\eta \frac{{I}_{m}Ak}{4{\rm{\pi }}{{r}_{m}}^{{\rm{2}}}}\,\sin \,{\theta }_{m}.$$

These components are all expressed in Cartesian coordinates to simplify the calculation of the Poynting vector through superposition of the electric field ***E*** = ***E***_1_ + ***E***_2_ and the magnetic field ***H*** = ***H***_1_ + ***H***_2_. From equation (3), ${\rm{Re}}({{\boldsymbol{E}}}_{1}\times {{\boldsymbol{H}}}_{1}^{\ast })$ and ${\rm{Re}}({{\boldsymbol{E}}}_{2}\times {{\boldsymbol{H}}}_{2}^{\ast })$ are both zero, irrespective of the nature of the terminal load, and thus only ${\rm{Re}}({{\boldsymbol{E}}}_{1}\times {{\boldsymbol{H}}}_{2}^{\ast })$ and ${\rm{Re}}({{\boldsymbol{E}}}_{2}\times {{\boldsymbol{H}}}_{1}^{\ast })$ need to be calculated. From equations (5–7), if the terminal load is an inductor or capacitor,

8 $${\rm{Re}}({{\boldsymbol{E}}}_{1}\times {{\boldsymbol{H}}}_{2}^{\ast })={\rm{Re}}({{\boldsymbol{E}}}_{2}\times {{\boldsymbol{H}}}_{1}^{\ast })=0.$$

However, if the terminal load is a resistor,

9 $${\rm{Re}}({{\boldsymbol{E}}}_{1}\times {{\boldsymbol{H}}}_{2}^{\ast })=\frac{{|{I}_{1}|}^{2}\eta F{A}^{2}k}{16{{\rm{\pi }}}^{{\rm{2}}}R{r}_{1}^{2}{r}_{2}^{3}}\,\sin \,{\theta }_{1}[(2\,{\cos }^{2}{\theta }_{2}-{\sin }^{2}{\theta }_{2}){{\boldsymbol{y}}}_{0}-1.5\,\sin \,2{\theta }_{2}{{\boldsymbol{z}}}_{0}],$$

10 $${\rm{Re}}({{\boldsymbol{E}}}_{2}\times {{\boldsymbol{H}}}_{1}^{\ast })=\frac{{|{I}_{1}|}^{2}\eta F{A}^{2}k}{16{{\rm{\pi }}}^{{\rm{2}}}R{r}_{1}^{3}{r}_{2}^{2}}\,\sin \,{\theta }_{2}[(2\,{\cos }^{2}{\theta }_{1}-{\sin }^{2}{\theta }_{1}){{\boldsymbol{y}}}_{0}-1.5\,\sin \,2{\theta }_{1}{{\boldsymbol{z}}}_{0}].$$

In terms of equation (8), the Poynting vector ***S*** = Re[(***E***_1_ + ***E***_2_) × (***H***_1_ + ***H***_2_)^*^]/2 of the superimposed field at an inductive or capacitive load at any point in the *yoz* plane between the two coils is zero. For any point in any other plane passing through the *z*-axis between the two coils, the result is obviously identical. Therefore, the net energy does not flow from the transmitting coil to the receiving coil, although the two coils are continuously involved in the process of charging and discharging of the electrical and magnetic energy. This conclusion meets our requirements because the capacitor and the inductor are energy storage elements that operate without consuming power.

In equations (9) and (10), the Poynting vector has two directions: one is the *y* direction, and the other is the *z* direction. In Fig. 1(b), for any point *p* in the *yoz* plane, there is always another point *q* that is symmetrical with it about the centre plane (*z* = *h*/2, not shown in Fig. 1(b)). The sum of the Poynting vectors of the superimposed field in the *y* direction at points *p* and *q* is zero. This is because the terms that contain *y* in equations (9) and (10) at points *p* and *q*, respectively, are opposite numbers, and the terms that contain *y* in equations (9) and (10) at points *q* and *p*, respectively, are opposite numbers. For any two other symmetric points in any other plane that passes through the *z*-axis between the two coils, a similar conclusion can be drawn. Therefore, the energy does not flow in the transverse directions.

In contrast, the Poynting vectors in the *z* direction in equation (9) and (10) are −1.5 sin 2*θ*_2_ and 1.5 sin 2*θ*_1_, respectively. These vectors are positive because *θ*_1_ ∈ (0°, 90°) and *θ*_2_ ∈ (90°, 180°); therefore, the energy flow density in the *z* direction at any point in the *yoz* plane between the two coils is nonzero. Additionally, we note that the energy flow density in the *z* direction has no relationship with the azimuth *φ*. The energy flow from the transmitting coil to the receiving coil can then be calculated using the surface integral of the Poynting vector over the centre plane (for any point in the plane, *θ*_1_ = *θ*_2_ = *θ* and *r*_1_ = *r*_2_ = *r*), and it can be simplified as

11 $$P=\int {\boldsymbol{S}}\cdot {\rm{d}}{\boldsymbol{A}}=\frac{1}{2}\int {\rm{Re}}({\boldsymbol{E}}\times {{\boldsymbol{H}}}^{\ast })\cdot {\rm{d}}{\boldsymbol{A}}=\frac{3{|{I}_{1}|}^{2}\eta F{A}^{2}k}{32{{\rm{\pi }}}^{{\rm{2}}}R}{\int }_{0}^{\infty }\frac{\sin \,\theta \,\sin \,2\theta }{{r}^{5}}\cdot 2{\rm{\pi }}x{\rm{d}}x=\frac{1}{2}{|{I}_{2}|}^{2}R,$$

where d*A* = 2π*x*d*x* is the differential area of the centre plane. Clearly, the result of equation (11) is correct because it is exactly equal to the power that is consumed by the load resistance; therefore, the conclusion that the essential aspect of wireless power transfer is superposition of the evanescent fields is completely correct.

### Electromagnetic and circuit simulations

**Figure 2:**
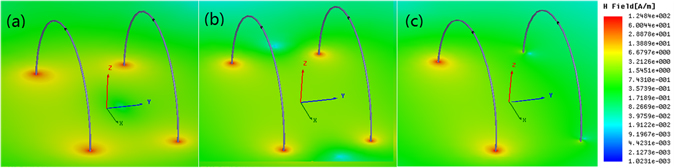
Incident and reflected magnetic field magnitude distributions. (**a**) The input port has a 10 Ω resistance, and the output has a 10 Ω inductive impedance. (**b**) The input port has a 10 Ω resistance, and the output has a 10 Ω capacitive reactance. (**c**) The input port has a 10 Ω resistance, and the output also has a 10 Ω resistance. The left and right coils are the transmitting and receiving coils, respectively. The black spots on the left and right of the coils are the input and output ports, respectively.

**Figure 3:**
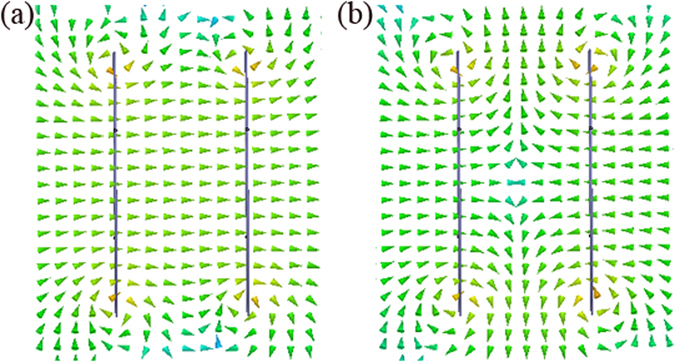
Incident and reflected magnetic field vector distributions in *xy* plane. (**a**) Distribution corresponding to Fig. 2(a). (**b**) Distribution corresponding to Fig. 2(b).

To validate the above conclusions, a physical model of the WPT system is set up using the commercial full-wave electromagnetic solver. In this model, the radius of the PEC coil with the cross-sectional radius *d* = 1 mm is *a* = 100 mm. An 80 pF chip capacitor is inserted into the coil to achieve resonance. An input port with power of 1 W and resistance of 10 Ω is set in the transmitting coil. The properties of the output port in the receiving coil and the transmission distance between the two coils can be set arbitrarily according to the transfer requirements. Our study results show that, irrespective of the magnitude of the transmission distance, the relationship between the reflected and incident fields is consistent with that of the theoretical analysis. This again indicates that the superposition principle of the evanescent fields is not confined to the condition where *a* ≪ *h*. For example, if the space between the two coils is 100 mm, then the simulation results at a given time are as shown in Figs 2(a–c) and 3(a and b). In Figs 2(a) and 3(a), we can see that the reflected and incident fields are out of phase when the terminal load is inductive; in Figs 2(b) and 3(b), the reflected and incident fields are in phase when the terminal load is capacitive; and in Fig. 2(c), we see that the phase of the reflected field lags behind that of the incident field by 90° when the terminal load is resistive.

**Figure 4:**
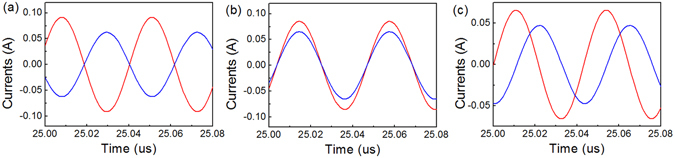
Time-domain current waveforms at resonant frequency. (**a**) Waveforms when the terminal load is an inductor of 68.6 nH. (**b**) Waveforms when the terminal load is a capacitor of 686 pF. (**c**) Waveforms when the terminal load is a resistor of 10 Ω. Red lines represent current *I*_1_, and blue lines represent current *I*_2_.

In contrast, we analysed the time-domain current waveforms of the equivalent circuit of the above physical model. The coil inductance of 590 nH is calculated from the formula^4^ *L* = *μ*_0_ *a*[ln(8*a*/*d*)−2]. The chip capacitance *C*_0_ is 80 pF, and thus the resonant frequency of both coils is 23.2 MHz. The mutual inductance between the two coils, which represents the transmission distance, is calculated from the formula^13^ $M=a{\mu }_{0}{\int }_{0}^{{\rm{\pi }}}(\cos \,\beta /\sqrt{2(1-\,\cos \,\beta )+{(h/a)}^{2}}){\rm{d}}\beta$. When the transmission distance is 100 mm, the mutual inductance is 49.4 nH. The electromotive force and the internal resistance of the power source are set at 1 V and 10 Ω, respectively. At the resonant frequency, the 10 Ω output port reactance values shown in Fig. 2(a) and (b) are actually equivalent to an inductor of 68.6 nH and a capacitor of 686 pF, respectively. The current waveform simulation results in the period from 25 to 25.08 μs are shown in Fig. 4(a–c). We see that the phase relationship between the two currents agrees very well with those obtained from the full-wave electromagnetic simulation results and the theoretical analysis. It should be noted that the power source of 1 V is not equivalent to 1 W in the electromagnetic model. Here, we mainly discuss the phase relationships between the two currents.

**Figure 5:**
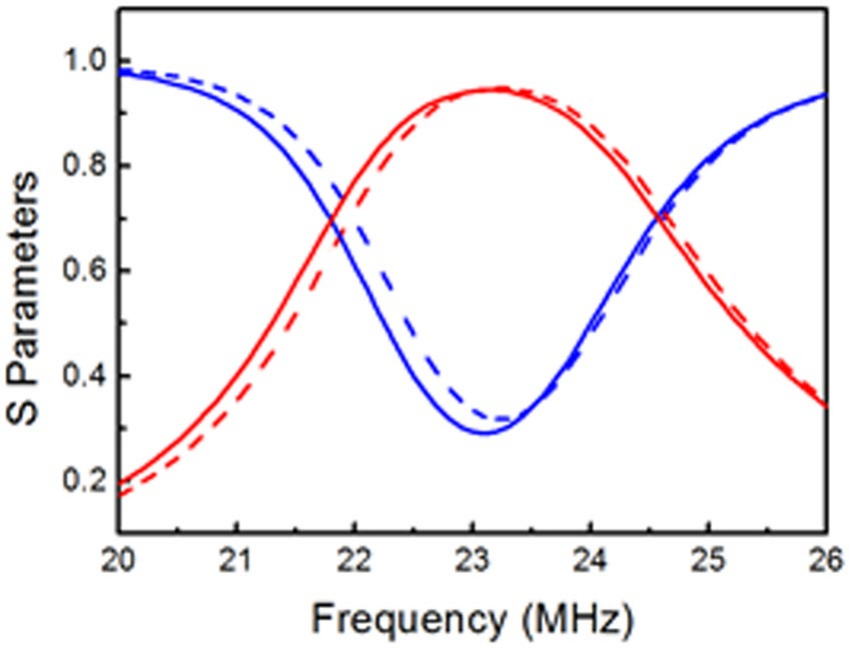
Scattering parameters. Solid and dashed lines represent the electromagnetic and circuit simulation results, respectively. The blue and red lines represent S_11_ and S_21_, respectively.

From the above analysis, we now know that the WPT system is equivalent to a two-port network, and thus we can use the reflection coefficient *S*_11_ and the transmission coefficient *S*_21_ to describe its performance. The results of the circuit and electromagnetic simulations are consistent (as shown in Fig. 5). This means that the extracted equivalent circuit parameters are correct. At the resonant frequency of 23.2 MHz, the transfer efficiency is very high, because the power that is carried by the superimposed field is absorbed completely by the resistive load.

## Discussion

It should be noted that Faraday’s law of electromagnetic induction plays an important role in the wireless power transfer process, because it guarantees the existence of the induced current in the receiving coil, which is a prerequisite for the superposition of the evanescent fields. However, it is not a sufficient condition for energy transfer. While the incident and reflected evanescent fields exist simultaneously at the different terminal loads, the superposition of these fields can only transmit energy (i.e., the energy is consumed) in the presence of a resistive load, which is the appeal of the nonradiative WPT when compared with microwave power transmission^15^. Using the theory of evanescent field superposition presented in this paper, the essence of the WPT process can be thoroughly explained in the electromagnetic domain, rather than in an equivalent circuit.

In addition, the electromagnetic field (wave) must be modulated to achieve certain specific goals. For example, magnetostatic energy has been harvested and distributed as desired in space to increase the sensitivity of magnetic sensors^16^, and the radiated energy in the far field has been concentrated in a narrow cone for directive emission^17^. Therefore, for nonradiative near-field energy transfer, the evanescent field should also be mediated to improve both transmission power and efficiency^18^ and to reduce the overall system size. In the process of understanding the essence of the WPT process and the characteristics of the evanescent field distributions, these problems will also be solved gradually.

## Methods

In our theoretical analysis, we used the point magnetic dipole model to discuss the incident and reflected evanescent field distributions. Through analysis of the Poynting vector of the superimposed field at the different terminal loads, the essence of the energy transmission process is clarified. The electromagnetic software was used to build a two-coil WPT system that was loaded with chip capacitances and contained an input/output port. The port characteristics and the WPT system size can be adjusted, and we can thus simulate a real physical WPT system model. From the physical model, equivalent circuit parameters are extracted. The electromagnetic simulation results and the time-domain current waveforms agree very well with the results of the theoretical analysis. These analyses have thus combined to prove that the essence of the WPT process is the superposition of the evanescent fields.
